# AOD Use and Attention Deficit/Hyperactivity Disorder

**Published:** 1998

**Authors:** Timothy E. Wilens

**Affiliations:** Timothy E. Wilens, M.D., is director of Substance Abuse Services in the Clinical Research Program in Pediatric Psychopharmacology at Massachusetts General Hospital and an associate professor of psychiatry at Harvard Medical School, Boston, Massachusetts

**Keywords:** attention deficit disorder, hyperactive behavior, child, AODD (alcohol and other drug use disorder), comorbidity, drug therapy, disease course, AODD recovery, self administration of drugs, biochemical mechanism, adulthood, AOD prevention, psychosocial treatment method, literature review

## Abstract

Children with attention deficit/hyperactivity disorder (ADHD) often continue to exhibit significant impairment in academic, occupational, and social functioning throughout adulthood. In addition, children with ADHD are at increased risk for developing alcoholism and other drug addictions, especially if alcoholism or ADHD exists in other family members. Alcohol and other drug (AOD) abuse may develop earlier in life (i.e., in midadolescence) when ADHD is accompanied by certain behavioral or mood disorders. The nature of the link between ADHD and AOD use disorder is unknown, although the association may be mediated by the co-occurring disorders just mentioned. In addition, ADHD-related AOD abuse may develop initially as an attempt to alleviate symptoms of mental distress associated with chronic failure, feelings of inadequacy, and conflict with parents and peers. Therapeutic intervention should incorporate both addiction and mental health treatment, including appropriate use of psychiatric medications.

Since the beginning of the century, terms such as “hyperactive” have been used to define a heterogeneous group of restless and inattentive children who display learning difficulties. Many such children exhibit the condition now known as attention deficit/hyperactivity disorder (ADHD). Children with ADHD may run about aimlessly, fidget and squirm when seated, and talk excessively. They are often irritable, impatient, and impulsive. Additional symptoms may include difficulty concentrating, forgetfulness, daydreaming, and easy distractibility. The disorder may persist into adulthood, accompanied by significant academic, occupational, and social impairment.

The presence of ADHD is an important risk factor for the development of alcoholism and other disorders associated with the use of alcohol and other drugs (AODs).^1^ In addition, AOD use disorders (AODDs) tend to appear earlier and to progress more rapidly in persons with ADHD. Alcoholics with ADHD also are less likely than those without ADHD to remain in alcoholism treatment programs or to achieve moderation or abstinence (Tarter and Edwards 1988). The identification of specific risk factors for AODD in persons with ADHD may permit more targeted prevention and treatment programs for both disorders at earlier stages of their development.

This article explores the co-occurrence (i.e., comorbidity) of ADHD and AODD and suggests possible mechanisms that may underlie their association. A discussion of ADHD treatment focuses on the use of medications that can alleviate symptoms of ADHD, thereby potentially affecting the subsequent development of AODD.

## Attention Deficit/Hyperactivity Disorder

The fourth revision of the *Diagnostic and Statistical Manual of Mental Disorders* (DSM–IV) (American Psychiatric Association [APA] 1994) presents criteria for establishing a diagnosis of ADHD. According to DSM–IV, behavioral symptoms of ADHD are divided into the categories of “inattention” and “hyperactivity-impulsivity.” Most patients (i.e., 75 percent) exhibit combined symptoms of inattention and hyperactivity-impulsivity. Between 15 and 20 percent of patients with ADHD exhibit only symptoms of inattention, and between 5 and 10 percent exhibit hyperactivity-impulsivity but not inattention (for a review, see Biederman 1998).

Additional DSM–IV criteria help distinguish ADHD from other disorders or normal temperamental traits. For example, the symptoms must be pervasive (e.g., occurring both at home and at work or in school) and persistent (e.g., not a transient reaction to stress) and must cause significant impairment in social, academic, or occupational functioning (APA 1994).^2^

ADHD affects from 6 to 9 percent of children and adolescents and up to 5 percent of adults (Biederman 1998). Data suggest that childhood ADHD persists into adolescence in approximately 75 percent of the patients and into adulthood in approximately 50 percent of the patients (Biederman 1998). The disorder may remit spontaneously in adolescence, although some symptoms, especially attention-related problems, may persist into adulthood.

## Extent of Comorbidity of ADHD and AODD

ADHD and AODD occur together in the same person, either simultaneously or sequentially, more often than would be expected by chance (Wilens et al. 1996). Symptoms of ADHD appear years before the development of AODD, often as early as infancy (APA 1994).

Studies have identified comorbid ADHD in 25 to 30 percent of certain adolescents with AODD. However, the subjects of the studies were repeat criminal offenders and patients in residential settings who were undergoing treatment for psychiatric and addictive disorders (Wilens et al. 1996). Consequently, the applicability of those results to the general population is uncertain. Among adults with AODD, rates of ADHD range from 15 to 25 percent (Wilens et al. 1996).^3^ Conversely, approximately 50 percent of adults with ADHD exhibit AOD abuse or dependence (Wilens et al. 1996). Data suggest that the risk of AODD developing at any time over the life span of an adult with ADHD is twice that of adults without ADHD (52 percent versus 27 percent, respectively) (Biederman et al. 1995).

Evidence from family studies confirms an association between co-occurring ADHD and AODD. Elevated rates of alcoholism are consistently found in parents of youth with ADHD (Wilens et al. 1996). Conversely, child and adolescent offspring of alcoholics, compared with children of nonalcoholics, exhibit lower attention spans; higher levels of impulsivity, aggressiveness, and hyperactivity; and elevated rates of ADHD (Wilens et al. 1996). Those data support the hypothesis that both disorders are familial and probably genetically transmitted.

The interpretation of family studies is complicated by the possible influence of prenatal exposure to AODs. For example, the offspring of alcohol- and cocaine-dependent mothers are at increased risk for psychiatric abnormalities, including ADHD (Wilens et al. 1996). Family genetic data in those studies are generally insufficient to determine the relative contributions of AOD exposure; parental psychopathology; and the interaction of genetic and environmental factors, such as the effects of poverty and poor prenatal care.

## Course and Remission

AODD appears at an earlier age among adults with, compared with those without, ADHD (mean age 19 versus 22) (Wilens et al. 1997). In addition, adults with AODD exhibit increased severity of AOD use problems when ADHD is also present (Carroll and Rounsaville 1993). The presence of ADHD appears to accelerate the transition from AOD abuse to dependence (Wilens et al. 1998) and increases the risk for developing a drug use disorder among subjects already abusing alcohol (Biederman et al. in press).

ADHD also affects the rate of recovery from AODD. In one study, adults with ADHD took more than twice as long as subjects without ADHD to recover from comorbid AODD (144 versus 60 months, respectively). In addition, AODD lasted more than 3 years longer in the subjects with ADHD (Wilens et al. 1998).

## Possible Mechanisms Linking ADHD and AODD

The underlying mechanisms that link ADHD to AODD remain unknown but may involve complex interactions among biological and psychological factors. Two possible contributory factors of particular significance to risk evaluation and treatment strategy are comorbidity with additional psychiatric disorders and the use of AODs to self-medicate psychological distress.

## Comorbid Psychiatric Disorders

Certain psychiatric conditions frequently co-occur in youth with combined ADHD and AODD (Wilens et al. 1996). Among those conditions are bipolar disorder and conduct disorder. Juvenile bipolar disorder is characterized by irritability and mood swings. Conduct disorder is characterized by a persistent pattern of aggressive behavior, criminality, or violation of social norms (APA 1994). Childhood ADHD is associated with the development of AODD in adolescence independent of other comorbid psychiatric conditions. However, the presence of conduct disorder or bipolar disorder confers a risk for an even younger onset of AODD (i.e., at age 16 or younger) independent of ADHD (Wilens et al. 1997; Biederman et al. 1997). Although anxiety and depressive disorders may co-occur with ADHD, they do not appear to confer additive risk for the development of AODD during adolescence in ADHD youth (Biederman et al. 1997).

## Self-Medication

Evidence suggests that some use of AODs represents in part an attempt to ameliorate psychiatric symptoms and their associated subjective states of distress (Khantzian 1997). The academic underachievement and behavioral problems associated with ADHD may result in conflicts with adults and peers, chronic failure, and demoralization. It is plausible that some people with ADHD may develop AODD in response to the emotional effects of social, occupational, and emotional impairment and accompanying poor self-image. Consistent with this theory, adolescents with ADHD report using AODs for mood adjustment rather than to achieve a “high” (Biederman et al. 1995; Wilens et al. 1996).

## Treatment Considerations

Treatment of patients with comorbid ADHD and AODD should be based on a thorough evaluation of relevant psychiatric, social, cognitive, educational, and family factors, along with a history of the patient’s AOD use and treatment. The clinician should rule out medical and neurological conditions whose symptoms may overlap with ADHD (e.g., the restlessness and emotional lability of hyperthyroidism) or result from AOD use (e.g., agitation associated with withdrawal) (Riggs 1998).

The treatment of ADHD and AODD must be considered simultaneously; however, if the addiction is active, it must be treated immediately (Riggs 1998). Depending on the severity and duration of the AODD, patients may require treatment in a residential facility. Psychosocial therapies aimed at co-occurring ADHD and AODD may be combined with patient and family education, attendance at self-help groups (e.g., Alcoholics Anonymous), and the use of appropriate medications (i.e., pharmacotherapy) (Riggs 1998).

### Pharmacotherapy

Medications play an important role in the long-term treatment of ADHD. The influence of such treatment on the subsequent development of AODD remains unclear and may depend in part on the severity of the underlying condition. Nevertheless, results of preliminary studies suggest that certain medications can alleviate ADHD symptoms in adolescents and adults while reducing co-occurring AOD use or cravings (Levin et al. 1997; Riggs 1998).

The most commonly used medications for treating ADHD are the psychostimulants, a class of medications that promote increased ability to concentrate, wakefulness, and alertness. Psychostimulants prescribed for ADHD include amphetamine (Dexedrine^®^, methylphenidate (Ritalin^®^), and pemoline (Cylert^®^) (Riggs 1998). Children with ADHD treated with those medications may exhibit increased ability to pay attention in class as well as improved academic and social performance.

Psychostimulants themselves are subject to abuse. Of the three psycho-stimulants just mentioned, pemoline has the lowest potential for abuse, followed by methylphenidate and amphetamine (Riggs 1998; Drug Enforcement Administration [DEA] 1995). The use of psychostimulants as prescribed for ADHD does not promote subsequent misuse of stimulants themselves or of other potentially addictive drugs (Hechtman 1985). In one study, patients with ADHD who were untreated and those who had poorer responses to psychostimulants exhibited more illegal AOD use than did patients treated successfully (Loney et al. 1981). However, patients with ADHD and their families should be instructed not to give psychostimulants to persons to whom they are not prescribed (DEA 1995).

Additional medications used to treat ADHD include various antidepressants (e.g., bupropion [Wellbutrin^®^], imipramine [Tofranil^®^ and others], and venlafaxine [Effexor^®^]) and certain medications commonly prescribed to treat high blood pressure (e.g., clonidine [Catapres^®^]) (Spencer et al. 1996). The choice of such medications depends in part on potential adverse interactions with AODs that the patient may be using. During the course of pharmacotherapy, the clinician should frequently monitor compliance with treatment, perform random urine analyses to detect AOD use, and coordinate treatment with other caregivers.

## Conclusions

Research supports a relationship between ADHD and AODD, with symptoms of ADHD appearing many years before the earliest onset of AOD abuse. AODDs that begin in adolescence run a more severe course than those that appear in adulthood. Therefore, prevention and early treatment strategies should be directed at children with ADHD before AOD use problems develop and become chronic (Wilens et al. 1997). More studies that follow disease development over the life span are needed to distinguish the relative contributions of causal factors linking these disorders and to identify youth at increased risk for ADHD–AODD comorbidity. This type of research may lead to more effective treatments, including pharmacotherapies, for both types of disorders (Biederman 1998).

## Footnotes

1 AOD disorders considered here include addiction (i.e., dependence) and abuse.

2 The term “ADHD” as used in this article also refers to previous definitions of the disorder.

3 By comparison, between 10 and 30 percent of adults in the United States exhibit alcohol use disorders (Kessler et al. 1997).
